# Atherogenic index of plasma and risk of cardiovascular disease among Cameroonian postmenopausal women

**DOI:** 10.1186/s12944-016-0222-7

**Published:** 2016-03-09

**Authors:** Jobert Richie N. Nansseu, Vicky Jocelyne Ama Moor, Murielle Elsa D. Nouaga, Bertrand Zing-Awona, Gladys Tchanana, Arthur Ketcha

**Affiliations:** Department of Public Health, Faculty of Medicine and Biomedical Sciences, University of Yaoundé I, PO Box 1364, Yaoundé, Cameroon; Sickle Cell Disease Unit, Mother and Child Centre, Chantal Biya Foundation, Yaoundé, Cameroon; School of Health Sciences, Catholic University of Central Africa, Yaoundé, Cameroon; Laboratory of Biochemistry, Yaoundé University Teaching Hospital, Yaoundé, Cameroon; Department of Physiological Sciences and Biochemistry, Faculty of Medicine and Biomedical Sciences, University of Yaoundé I, Yaoundé, Cameroon; Mathematical Engineering and Information System Laboratory, National Advanced School of Engineering, University of Yaoundé I, Yaoundé, Cameroon; African Center of Excellence in Information and Communication Technologies, Yaoundé, Cameroon

**Keywords:** Atherogenic index of plasma, Menopause, Cardiovascular disease, Cameroon

## Abstract

**Background:**

The paucity of data regarding the relationship between atherogenic index of plasma (AIP) and risk of cardiovascular disease (CVD) in postmenopausal women living in sub-Saharan Africa prompted us to conduct this study which aimed at assessing the interplay between AIP and risk of CVD among Cameroonian postmenopausal women.

**Methods:**

This was a cross-sectional study conducted among 108 postmenopausal women in Yaoundé, Cameroon. Risk of CVD was calculated using the Framingham risk score, (FRS), and the AIP was derived as log (triglycerides/high-density lipoproteins cholesterol).

**Results:**

Mean age of participants equaled 56.4 ± 6.9 years. AIP values ranged from -0.40 to 0.85 with a mean of 0.21 ± 0.27. There was a positive and significant correlation between AIP and body mass index (*r* = 0.234; *p* = 0.015), systolic blood pressure (*r* = 0.350; *p* < 0.001), diastolic blood pressure (*r* = 0.365; *p* < 0.001), fasting plasma glucose (*r* = 0.314; *p* = 0.001), uric acid (*r* = 0.374; *p* < 0.001), and total cholesterol (*r* = 0.374; *p* < 0.001), but not with age (*r* = -0.104; *p* = 0.284). The FRS varied between 1.2 % and >30 % with a mean of 13.4 ± 8.7 %. In univariable model, AIP significantly influenced the risk of CVD (β = 11.94; *p* < 0.001; R^2^ = 0.136). But in the multivariable model, after adjusting for confounders, AIP did not impact the risk of CVD anymore (adjusted β = 1.98; *p* = 0.487; R^2^ = 0.486).

**Conclusion:**

AIP may not be an independent factor impacting the risk of CVD among Cameroonian postmenopausal women. More studies are needed to better elucidate the interaction between AIP and risk of CVD in our setting.

## Background

Menopause, which is the permanent cessation of menstruation following loss of ovarian activity, has considerable impact on social, reproductive, physical and psychological health of the woman [1]. While premenopausal women have a lower incidence of cardiovascular disease (CVD) compared with men of the same age, the incidence of the disease in women increases dreadfully after the age of 50 years [1]. The anti-atherogenic effect of estrogens and the protection of females against CVD, especially coronary heart disease are well described during the premenopausal period [2]. Indeed, there is convincing evidence that menopause is associated with a pro-atherogenic lipid profile characterized by lower high-density lipoproteins cholesterol (HDL-C), higher low-density lipoproteins cholesterol (LDL-C) and triglyceride (TG) levels [3], central adiposity [4], increased diastolic blood pressure [5] and increased insulin resistance [6], hence an increased likelihood to develop CVD.

A variety of indices have been used for the diagnosis and prognosis of CVD. In this regard, some researchers have looked at the relationship between TG and HDL-C, and have shown for instance that the ratio of TG to HDL-C was a strong predictor of myocardial infarction [7]. There have been some claims that the atherogenic index of plasma (AIP), which is the logarithmic transformation of the just-mentioned ratio (TG/HDL-C), could be used as a significant predictor of atherosclerosis [8], and CVD as well [9–11]. Dobiásová M suggested indeed that AIP values of -0.3 to 0.1 may be associated with low, 0.1 to 0.24 with medium and above 0.24 with high risk of CVD [10]. It was bolstered that the strong correlation of AIP with lipoprotein particle size may explain its high predictive value of CVD occurrence [8].

However, there is paucity of data scrutinizing the interplay between AIP and risk of CVD in postmenopausal women living in Africa, especially south of the Sahara. We therefore undertook the present study, which aimed at investigating the link between AIP and risk of CVD among Cameroonian postmenopausal women.

## Methods

### Study design and participants

In January 2015, we conducted a cross-sectional study at the Yaoundé University Teaching Hospital, Cameroon. We recruited postmenopausal women whose last menses dated back to at least 1 year, with no previous history of cardiovascular events, in apparent good health, who were visiting the study site for various reasons, and who voluntarily accepted to take part in the survey. A convenient sample of 100 women was set, and women fulfilling our inclusion criteria were enrolled during the study period. Part of the methods is also reported elsewhere [12].

### Data collection

We used a structured pre-tested questionnaire for data collection, including socio-demographic characteristics (age, region of origin, profession, marital status), medical history (past medical events, family history of diabetes, hypertension or other relevant disease), and lifestyle habits (physical exercise, smoking (defined as having smoked any cigarettes in the past month), consumption of alcohol, and consumption of fruits and vegetables). Afterwards, a physical examination was undertaken during which blood pressure (composite of systolic blood pressure (SBP) and diastolic blood pressure (DBP) in mmHg), body weight (to the nearest kilogram), and height (to the nearest centimeter) were measured. Body mass index (BMI) was subsequently derived as weight (kg)/height × height (m). At the end of this stage, participants were given an appointment for blood collection.

### Blood sampling and biochemical measurements

Fasting blood samples were collected after a 12-h overnight fasting, and processed for biochemical determinations including fasting plasma glucose (FPG), total cholesterol (TC), triglycerides (TG), high-density lipoproteins cholesterol (HDL-C) and uric acid. Low-density lipoproteins cholesterol (LDL-C) was then calculated using Friedwald’s formula [13]. The very low-density lipoproteins cholesterol (VLDL-C) was derived based on the formula: VLDL-C = TG/2.2 [14]. The atherogenic index of plasma (AIP) was calculated as the logarithmically transformed ratio of molar concentrations of TG to HDL-C [8].

### Cardiovascular risk assessment and stratification

The Framingham risk score (FRS) was used to measure the 10-year risk of cardiovascular events [15], and was electronically calculated after entering the required parameters (age, sex, diabetes (yes/no), smoking (yes/no), BP lowering medications (yes/no), SBP, TC and HDL-C) on this web page: https://www.cvdriskchecksecure.com/FraminghamRiskScore.aspx/. Actually, the FRS predicts the 10-year risk of developing a coronary event (composite of myocardial infarction and coronary death), and is considered a standard and generally acceptable approach to risk prediction [16]. Participants were subsequently grouped into three classes, given they presented a low risk (score < 10 %), a moderate risk (score 10–20 %) or a high risk of CVD (score > 20 %) [15].

### Statistical analysis

Data were coded, entered and analyzed using SPSS version 20.0 (IBM SPSS Inc., Chicago, Illinois, USA). Results are presented as count (proportion) or mean ± standard deviation (SD) where appropriate. The Student *t* test or its nonparametric equivalent (Wilcoxon rank sum) served for comparison of quantitative variables. The Pearson correlation test was used to seek linear relation between quantitative variables. Linear regression analysis served to assess the impact of AIP on CVR by both univariable and multivariable models (while adjusting for confounders in a backward stepwise procedure). A *p* value < 0.05 was used to characterize statistically significant results.

### Ethical considerations

Before starting the study, an authorization was obtained from authorities of the Yaoundé University Teaching Hospital, and an ethical clearance was delivered by the Ethical Review Board of the Faculty of Medicine and Biomedical Sciences of the University of Yaoundé I, Cameroon. All the procedures used in the present study were in keeping with the current revision of the Helsinki Declaration. All participants were informed of the various aspects of the study, and they were enrolled only after providing a signed consent form.

## Results

We recruited 108 women, their ages varying between 45 and 80 years with a mean age of 56.4 ± 6.9 years (Table 1). Sixteen women (14.8 %) were known diabetes patients, and 37 (34.3 %) were known hypertensive. Five women (4.6 %) were active smokers. Thirty six women (33.3 %) regularly practiced a physical exercise (≥3 sessions per week), and 49 (45.4 %) were regularly consuming fruits and vegetables (i.e. at least thrice weekly).

**Table 1:** Distribution of clinical and biochemical parameters

Variable	Minimum	Maximum	Mean ± SD	Median (IQR)
Age	45	80	56.4 ± 6.9	56 (51.3–60)
BMI (kg/m^2^)	21.20	43.60	31.56 ± 5.27	31.6 (27.93–35.0)
Systolic BP (mmHg)	100.0	210.0	138.6 ± 23.6	140 (120–150)
Diastolic BP (mmHg)	60.0	120.0	80.2 ± 13.0	80 (70–90)
Uric acid (mg/l)	23.30	121.40	57.32 ± 18.19	53.9 (46.17–65.38)
FPG (g/l)	0.6	3.31	0.65 ± 0.30	0.79 (0.70–0.95)
TC (mmol/l)	2.72	10.96	5.92 ± 1.62	5.93 (4.75–6.83)
LDL-C (mmol/l)	0.57	9.17	4.11 ± 1.51	4.17 (3.15–4.89)
HDL-C (mmol/l)	0.54	2.28	1.25 ± 0.30	1.22 (1.04–1.42)
Triglycerides (mmol/l)	0.57	6.32	2.24 ± 1.22	2.01 (1.33–2.89)
VLDL-C (mmol/l)	0.26	2.87	1.02 ± 0.55	0.91 (0.61–1.32)
AIP	-0.40	0.85	0.21 ± 0.27	0.20 (0.00–0.38)
FRS (%)	1.2	>30	13.37 ± 8.67	11.7 (6.3–18.51)

*SD* standard deviation, *IQR* interquartile range, *BMI* body mass index, *BP* blood pressure, *FPG* fasting plasma glucose, *TC* Total Cholesterol, *LDL-C* low-density lipoproteins cholesterol, *HDL-C* high-density lipoproteins cholesterol, *VLDL-C* very low-density lipoproteins cholesterol, *AIP* atherogenic index of plasma, *FRS* Framingham risk score

The distribution of various clinical and biological variables measured is depicted by Table 1. AIP values ranged from -0.40 to 0.85 with a mean of 0.21 ± 0.27. The distribution of AIP was comparable between participants aged ≤ 60 years and their counterparts (*p* = 0.188). Likewise, AIP values did not vary significantly with regard to known diabetes (*p* = 0.511), known hypertension (*p* = 0.213), active smoking (*p* = 0.972), regular physical activity (*p* = 0.642), and regular consumption of fruits/vegetables (*p* = 0.154). AIP was positively and significantly correlated with BMI (*r* = 0.234; *p* = 0.015), SBP (*r* = 0.350; *p* < 0.001), DBP (*r* = 0.365; *p* < 0.001), FPG (*r* = 0.314; *p* = 0.001), uric acid (*r* = 0.374; *p* < 0.001), and TC (*r* = 0.374; *p* < 0.001), but not with age (*r* = -0.104; *p* = 0.284; Table 2).

**Table 2:** Correlation between the atherogenic index of plasma and other variables

Variable	Coefficient	*p* value
Age	-0.104	0.284
BMI	0.234	0.015^*^
Systolic BP	0.350	<0.001^*^
Diastolic BP	0.365	<0.001^*^
FPG	0.314	0.001^*^
Uric acid	0.374	<0.001^*^
TC	0.374	<0.001^*^
FRS	0.369	<0.001^*^

*BMI* body mass index, *BP* blood pressure, *FPG* fasting plasma glucose, *TC* Total Cholesterol, *LDL-C* low-density lipoproteins cholesterol, *FRS* Framingham risk score

^*^ *p* value < 0.05

The 10-year risk of cardiovascular events based on the FRS varied between 1.2 % and >30 % with a mean of 13.4 ± 8.7 % (Table 1). Forty three participants (39.8 %) had a low risk of CVD (<10 %), 39 women (36.1 %) had a moderate risk (10–20 %), and 21 women (24.1 %) had a high risk of CVD (>20 %). The AIP and the FRS were significantly correlated with each other (*r* = 0.369, *p* < 0.001; Table 2). Furthermore, there was a significant relation between the FRS (as the dependent variable) and the AIP in univariable linear regression analysis (β = 11.94, 95 % confidence interval (CI): 6.15–17.72; *p* < 0.001; R^2^ = 0.136).

In multivariable linear regression analysis after adjusting for BMI, FPG, uric acid, DBP, and LDL-C, AIP did not impact the risk of CVD anymore, even when BMI and uric acid were removed from the model. The adjusted β coefficient for AIP in this last model (adjusted for FPG, DBP and LDL-C) was 1.98 (95 % CI: -3.65–7.61; *p* = 0.487). Of note, FPG (adjusted β = 4.95, 95 % CI: 0.45–9.46; *p* = 0.032), DBP (adjusted β = 3.37, 95 % CI: 2.31–4.42; *p* < 0.001) and LDL-C (adjusted β = 1.17, 95 % CI: 0.26–2.07; *p* = 0.012) were independent factors impacting our women’s risk of CVD. These results are extensively presented in another report [12].

## Discussion

Results from this study showed that AIP and the risk of CVD (assessed by the FRS) were significantly correlated with each other, though this correlation was weak (*r* = 0.369). Furthermore, after adjusting for confounders, AIP seemed not to be an independent factor impacting the onset of CVD (*p* = 0.487).

We obtained higher AIP values (mean 0.21 ± 0.27) than Nwagha et al. [17] in their group of Nigerian postmenopausal women (mean 0.15 ± 0.35). Intriguingly, AIP was not influenced by our women’s age, and thus the duration of menopause, which concurs with results from Nwagha et al. [17]. Indeed, although these authors found that AIP levels significantly increased from before menopause to 10 years post menopause and 20 years post menopause (*p* < 0.0001), they did not show any significant difference between 10 and 20 years post menopause (*p* = 0.116) [17].

Likewise, we found no relation between AIP and known history of diabetes, in contradiction with previous reports [10, 18]. In the same line, AIP was uninfluenced by regular physical exercise and regular consumption of fruits/vegetables although these healthy lifestyle interventions have been shown to reduce TG levels and increase HDL-C titers [19–25]. As AIP is derived from the ratio of TG to HDL-C, it is expected that any action leading to a reduction in TG and/or an increase in HDL-C would result in consequential reduction in AIP. These contradictory results we obtained may be due to the lack of power or misreporting of information by our participants, as the reliability of data collected relied solely on their declaration.

AIP was positively and significantly correlated with BMI, SBP, DBP, FPG, uric acid, and TC which have been cited as risks factors for CVD [3–6, 18, 26–29]. Some authors have demonstrated for instance a close relation between AIP and plasma uric acid [18, 26], especially in women [26]. Therefore, we can guess that interventions to lessen the above parameters would perhaps lead to the diminution in AIP levels. As such, our postmenopausal women should be encouraged to adopt healthy lifestyles, as evidence has accumulated a beneficial effect on these parameters [19–25, 30]. Besides, some pharmacological interventions (such as hormone replacement therapy and vitamin D supplementation) could be considered, though most of them have yielded controversial outcomes [31].

We also found a positive and significant correlation between AIP and the risk of CVD. Based on our results, we found that API values < 0.163 (45 women) could have been associated with low, 0.163–0.273 (24 women) with moderate, and above 0.273 (38 women) with high risk of CVD. Mirroring our findings, Dobiásová M found that AIP values increased with increasing cardiovascular risk, and suggested that AIP values of -0.3 to 0.1 may be associated with low, 0.1 to 0.24 with medium and above 0.24 with high risk of CVD [10]. However, the relation we observed between AIP and the risk of CVD was weak, given that only 14 % of the variation of the FRS could be explained by the AIP (R^2^ = 0.136). Moreover, after adjusting for BMI, FPG, uric acid, DBP, and LDL-C, the association between AIP and the risk of CVD became unlikely. These results do not corroborate those from Dobiásová M where AIP, adjusted for age, BMI, waist circumference, type 2 diabetes, blood pressure, smoking, TG, TC, LDL-C, apolipoproteins B, HDL-C, and TC/HDL-C, was the best independent driver of positive findings at coronary angiography [10].

Our inability to find an independent influence of AIP on the risk of CVD could perhaps find an explanation in the lack of power, as the study was a cross-sectional one with a relative small sample size. Another limitation of this study could be the fact that, in the absence of locally-developed tools to assess the cardiovascular risk, we used the FRS which may lack to accurately assess the cardiovascular risk in African populations as they may present different patterns of CVD when compared with developed countries [32]. Furthermore, enrolment of participants in a hospital setting rather than in the community could have perhaps led to an overestimation of their risk of CVD. Larger, community-based and well-designed studies are therefore warranted in our milieu to better investigate the relationship between AIP and risk of CVD, especially among postmenopausal women. Besides, other studies are required to further assess whether AIP could be used as a valuable tool to predict the risk of CVD.

## Conclusion

This study conducted among 108 apparently healthy Cameroonian postmenopausal women showed a significant correlation between the atherogenic index of plasma and BMI, SBP, DBP, FPG, uric acid, and TC, but not with age, though these correlations were weak. However, AIP was not an independent factor impacting the risk of CVD. More studies are needed to better elucidate the interplay between AIP and risk of CVD in our setting.

## Abbreviations

AIP: atherogenic index of plasma
BMI: body mass index
CI: confidence interval
CVD: cardiovascular disease
DBP: diastolic blood pressure
FPG: fasting plasma glucose
FRS: Framingham risk score
HDL-C: high-density lipoproteins cholesterol
LDL-C: low-density lipoproteins cholesterol
SBP: systolic blood pressure
TC: total cholesterol
TG: triglycerides
VLDL-C: very low-density lipoproteins cholesterol

## References

[1] Dosi R, Bhatt N, Shah P, Patell R (2014). Cardiovascular disease and menopause. J Clin Diagn Res.

[2] Pahwa MB, Seth S, Seth RK (1998). Lipid profile in various phases of menstrual cycle and its relationship with percentage plasma volume changes. Clin Chim Acta.

[3] Warren MP, Halpert S (2004). Hormone replacement therapy: controversies, pros and cons. Best Pract Res Clin Endocrinol Metab.

[4] Augoulea A, Mastorakos G, Lambrinoudaki I, Christodoulakos G, Creatsas G (2005). Role of postmenopausal hormone replacement therapy on body fat gain and leptin levels. Gynecol Endocrinol.

[5] Reckelhoff JF (2004). Basic research into the mechanisms responsible for postmenopausal hypertension. Int J Clin Pract Suppl.

[6] Wu SI, Chou P, Tsai ST (2001). The impact of years since menopause on the development of impaired glucose tolerance. J Clin Epidemiol.

[7] Gaziano JM, Hennekens CH, O’Donnell CJ, Breslow JL, Buring JE (1997). Fasting triglycerides, high-density lipoprotein, and risk of myocardial infarction. Circulation.

[8] Dobiásová M, Frohlich J (2001). The plasma parameter log (TG/HDL-C) as an atherogenic index: correlation with lipoprotein particle size and esterification rate in apoB-lipoprotein-depleted plasma (FER(HDL)). Clin Biochem.

[9] Bakry OA, El Farargy SM, Ghanayem N, Soliman A. Atherogenic index of plasma in non-obese women with androgenetic alopecia. Int J Dermatol. 2015, doi: 10.1111/ijd.12783. [Epub ahead of print]. 10.1111/ijd.12783 26096895

[10] Dobiásová M (2006). AIP--atherogenic index of plasma as a significant predictor of cardiovascular risk: from research to practice [Article in Czech. Vnitr Lek.

[11] Soška V, Jarkovský J, Ravčuková B, Tichý L, Fajkusová L, Freiberger T (2012). The logarithm of the triglyceride/HDL-cholesterol ratio is related to the history of cardiovascular disease in patients with familial hypercholesterolemia. Clin Biochem.

[12] Ama Moor VJ, Nansseu JR, Nouaga ME, Noubiap JJ, Nguetsa GD, Tchanana G, Ketcha A, Fokom-Domgue J: Assessment of the 10-year risk of cardiovascular events among a group of sub-Saharan African post-menopausal women. Cardiol J 2015, [Epub ahead of print]. 10.5603/CJ.a2015.0056 26412602

[13] Friedewald WT, Levy RI, Fredrickson DS (1972). Estimation of the concentration of low-density lipoprotein cholesterol in plasma, without use of the preparative ultracentrifuge. Clin Chem.

[14] Crook MA. Plasma lipids and Lipoproteins. In: Clinical Chemistry and Metabolic Medicine. vol. 7th edition. London UK: Edwarld Arnold publishers Ltd; 2006:198–213.

[15] Wilson PW, D’Agostino RB, Levy D, Belanger AM, Silbershatz H, Kannel WB (1998). Prediction of coronary heart disease using risk factor categories. Circulation.

[16] D’Agostino RB, Grundy S, Sullivan LM, Wilson P, CHD Risk Prediction Group (2001). Validation of the Framingham coronary heart disease prediction scores: results of a multiple ethnic groups investigation. JAMA.

[17] Nwagha UI, Ikekpeazu EJ, Ejezie FE, Neboh EE, Maduka IC (2010). Atherogenic index of plasma as useful predictor of cardiovascular risk among postmenopausal women in Enugu, Nigeria. Afr Health Sci.

[18] Akbas EM, Timuroglu A, Ozcicek A, Ozcicek F, Demirtas L, Gungor A, Akbas N. Association of uric acid, atherogenic index of plasma and albuminuria in diabetes mellitus. Int J Clin Exp Med. 2014;7(12):5737–43. PMC4307547 25664100

[19] Davis J, Murphy M, Trinick T, Duly E, Nevill A, Davison G (2008). Acute effects of walking on inflammatory and cardiovascular risk in sedentary post-menopausal women. J Sports Sci.

[20] Durstine JL, Haskell WL (1994). Effects of exercise training on plasma lipids and lipoproteins. Exerc Sport Sci Rev.

[21] Pronk NP (1993). Short term effects of exercise on plasma lipids and lipoproteins in humans. Sports Med.

[22] Kazlauskaite R, Powell LH, Mandapakala C, Cursio JF, Avery EF, Calvin J (2010). Vitamin D is associated with atheroprotective high-density lipoprotein profile in postmenopausal women. J Clin Lipidol.

[23] Abedi P, Lee MH, Kandiah M, Yassin Z, Shojaeezade D, Hosseini M, Malihi R. Diet intervention to improve cardiovascular risk factors among Iranian postmenopausal women. Nutr Res Pract. 2010;4(6):522–7. 10.4162/nrp.2010.4.6.522 PMC3029794 21286411

[24] Song R, Ahn S, So HY, Park IS, Kim HL, Joo KO, Kim JS. Effects of Tai Chi exercise on cardiovascular risk factors and quality of life in post-menopausal women [Article in Korean]. J Korean Acad Nurs. 2009;39(1):136–44. 10.4040/jkan.2009.39.1.136 19265320

[25] Hardin DS, Azzarelli B, Edwards J, Wigglesworth J, Maianu L, Brechtel G, Johnson A, Baron A, Garvey WT. Mechanisms of enhanced insulin sensitivity in endurance-trained athletes: effects on blood flow and differential expression of GLUT 4 in skeletal muscles. J Clin Endocrinol Metab. 1995;80:2437–46. 10.1210/jcem.80.8.7629239 7629239

[26] Lippi G, Montagnana M, Luca Salvagno G, Targher G, Cesare Guidi G (2010). Epidemiological association between uric acid concentration in plasma, lipoprotein(a), and the traditional lipid profile. Clin Cardiol.

[27] Kim HC, Greenland P, Rossouw JE, Manson JE, Cochrane BB, Lasser NL, Limacher MC, Lloyd-Jones DM, Margolis KL, Robinson JG. Multimarker prediction of coronary heart disease risk: the Women’s Health Initiative. J Am Coll Cardiol. 2010;55(19):2080–91. 10.1016/j.jacc.2009.12.047 20447530

[28] Yusuf S, Reddy S, Ounpuu S, Anand S (2001). Global burden of cardiovascular diseases: part I: general considerations, the epidemiologic transition, risk factors, and impact of urbanization. Circulation.

[29] Sekuri C, Eser E, Akpinar G, Cakir H, Sitti I, Gulomur O, Ozcan C. Cardiovascular disease risk factors in post-menopausal women in West Anatolia. Jpn Heart J. 2004;45(1):119–31. 10.1536/jhj.45.119 14973357

[30] Kolovou G, Marvaki A, Bilianou H (2011). One more look at guidelines for primary and secondary prevention of cardiovascular disease in women. Arch Med Sci.

[31] Parazzini F (2014). Resveratrol, inositol, vitamin D and K in the prevention of cardiovascular and osteoporotic risk: a novel approach in peri- and postmenopause. Minerva Ginecol.

[32] GBD 2013 Mortality and Causes of Death Collaborators (2015). Global, regional, and national age-sex specific all-cause and cause-specific mortality for 240 causes of death, 1990-2013: a systematic analysis for the Global Burden of Disease Study 2013. Lancet.

